# Quercetin, Epigallocatechin Gallate, Curcumin, and Resveratrol: From Dietary Sources to Human MicroRNA Modulation

**DOI:** 10.3390/molecules25010063

**Published:** 2019-12-23

**Authors:** Erika Cione, Chiara La Torre, Roberto Cannataro, Maria Cristina Caroleo, Pierluigi Plastina, Luca Gallelli

**Affiliations:** 1 Department of Pharmacy, Health and Nutritional Sciences, Department of Excellence 2018–2022, University of Calabria, Edificio Polifunzionale, 87036 Rende (CS), Italy; erika.cione@unical.it (E.C.); latorre.chiara@libero.it (C.L.T.); r.cannataro@gmail.com (R.C.); mariacristinacaroleo@virgilio.it (M.C.C.); pierluigi.plastina@unical.it (P.P.); 2 Department of Health Science, School of Medicine, University of Magna Graecia, Clinical Pharmacology Unit, Mater Domini Hospital, 88100 Catanzaro, Italy

**Keywords:** nutrients, antioxidants, common use, phenolic compounds

## Abstract

Epidemiologic studies suggest that dietary polyphenol intake is associated with a lower incidence of several non-communicable diseases. Although several foods contain complex mixtures of polyphenols, numerous factors can affect their content. Besides the well-known capability of these molecules to act as antioxidants, they are able to interact with cell-signaling pathways, modulating gene expression, influencing the activity of transcription factors, and modulating microRNAs. Here we deeply describe four polyphenols used as nutritional supplements: quercetin, resveratrol, epigallocatechin gallate (ECGC), and curcumin, summarizing the current knowledge about them, spanning from dietary sources to the epigenetic capabilities of these compounds on microRNA modulation.

## 1. Introduction

**Figure 1:**
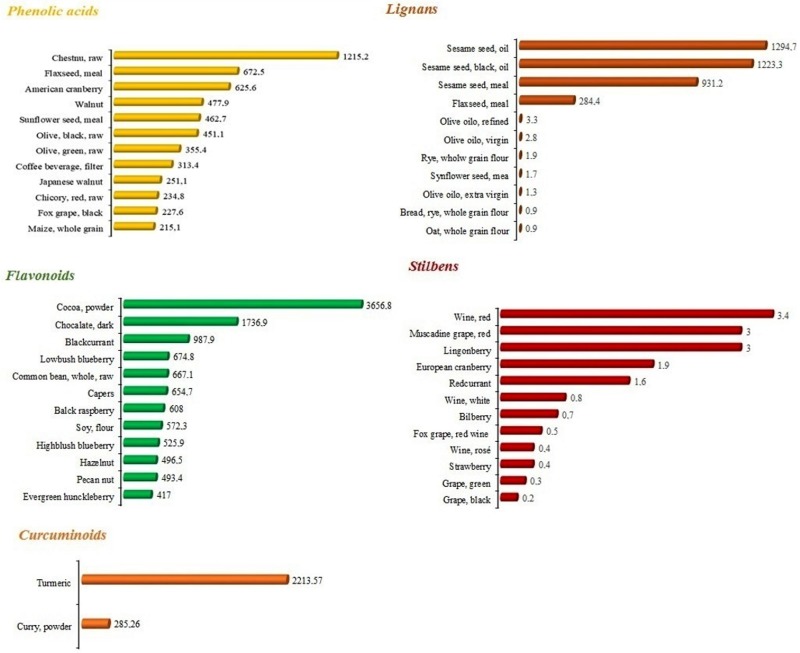
Content of phenolic acids, stilbenes, flavonoids, lignans, and curcuminoids (expressed as mg/100 g of food) in the main food sources of these polyphenolic classes, according to the database Phenol-Explore (http://phenol-explorer.eu/).

Epidemiologic studies suggest that dietary polyphenol intake is associated with a lower incidence of several non-communicable diseases including type 2 diabetes and cardiovascular disease (CVD) [1]. They are often linked to excessive production of reactive oxygen species (ROS) [2,3]. Polyphenols are the most abundant antioxidants in our diet and are commonly present in fruits [4], vegetables, cereals, olives [5], dry legumes [6], licorice [7], chocolate and beverages, such as tea, coffee, and wine. Divided into different classes, according to their chemical structure, polyphenols describe essentially phenolic acids, stilbenes, flavonoids, lignans, and curcuminoids (see Figure 1). Besides the well-known capability of these molecules to act as antioxidants, they are able to interact with cell-signaling pathways, modulating gene expression in two different ways: i) influencing the activity of transcription factors and ii) epigenetically, modulating microRNAs. Here we deeply describe four polyphenols used as nutritional supplements: quercetin, epigallocatechin gallate (ECGC), curcumin, and resveratrol, summarizing the current knowledge about them, spanning from dietary sources to their epigenetic capabilities.

## 2. Polyphenols

### 2.1. Dietary Sources

Polyphenols, mainly flavonoids, are secondary plant metabolites contained in fruits and vegetables [8]. Some of them are specific of particular foods, such as flavanones in citrus fruits [9], isoflavones in soy, and phloridzin in apples. On the other hand, other polyphenols, such as quercetin, are found in a plethora of vegetable products [10]. Biochemical and chemical activities of polyphenols have been tested with different anti-inflammatory and antioxidant methods [9,11]. Although, several foods contain complex mixtures of polyphenols, numerous factors such climate (sun exposure, precipitation) and/or agronomy and storage as well as maturity at the time of harvest, can affect their content in plants. Furthermore, simply peeling fruits or vegetables can significantly reduce polyphenolic content, since these substances are often present in high concentrations in the external parts.

#### 2.1.1. Phenolic Acids

Phenolic acids are derived from two main phenolic compounds: benzoic and cinnamic acids. Examples of hydroxybenzoic derivatives are gallic, vanillic, and syringic acids, whereas caffeic, ferulic, sinapic, and *p*-coumaric acids belong to hydroxycinnamic acids [12,13]. Fruits and vegetables are characterized mainly by the presence of free phenolic acids, whereas grains and derivatives by bound phenolic acids [14]. Hydroxycinnamic acids are present at high concentrations in fruits, vegetables, tea, cocoa, wine, coffee, and whole grains [15]. They exist either in free or conjugated form in plants [16]. The great interest in phenolic acids is associated with their use in food technology due to their high potential in food preservation [17].

#### 2.1.2. Lignans

**Figure 2:**
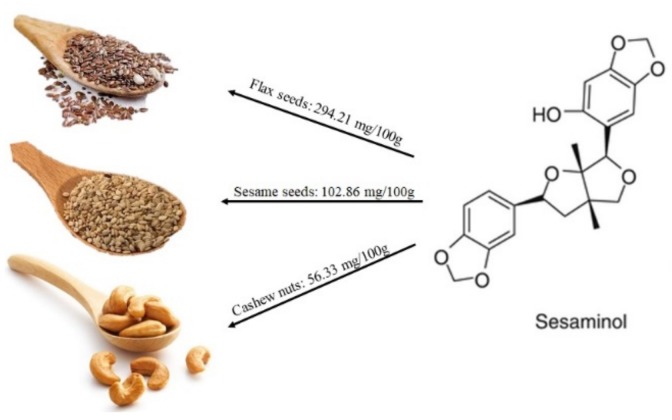
Main foods containing lignans, mostly sesaminol, according to the database Phenol-Explore (http://phenol-explorer.eu/).

Lignans are present in a wide variety of plant foods, including seeds (flax, pumpkin, sunflower, poppy, sesame), whole grains (rye, oats, barley), bran (wheat, oat, rye), beans, fruit (berries in particular), and vegetables [18,19,20]. Among edible plant components, the most concentrated lignan sources are sesame and flax seeds. Sesame seeds exhibit the second highest concentration of sesaminol followed by cashew nuts (see Figure 2). Regarding vegetables, the *Brassica* family contains pinoresinol, while spinach, white potatoes, and mushrooms contain low amounts of lignin [21,22,23]. Secoisolariciresinol and matairesinol were the first lignans identified in foods [24,25]. A variety of factors could affect lignan contents in plants, including geographic location, climate, maturity, and storage conditions.

#### 2.1.3. Flavonoids

**Figure 3:**
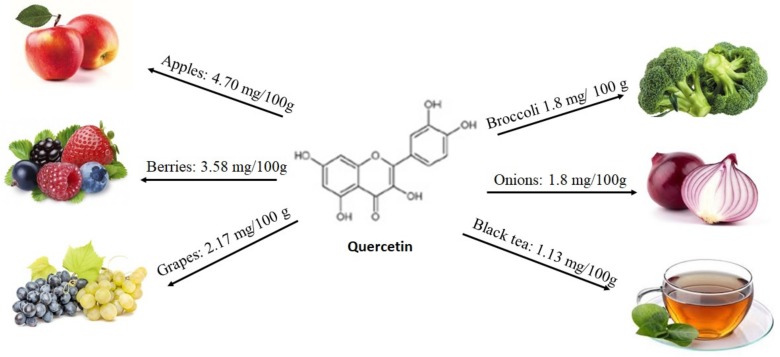
Foods that contains quercetin, according to the database Phenol-Explore (http://phenol-explorer.eu/).

Among several flavonoids present in foods, quercetin (a flavonol) and epigallocatechin-3-gallate (a flavanol) gained our scientific interest. Quercetin primarily enters into the diet as quercetin-3-glucoside (isoquercetin). This is hydrolyzed in the small intestine and is rapidly absorbed. Foods rich in quercetin include principally apples, berries, grapes, but also red onions, broccoli, black tea, green tea, pepper, red wine, tomatoes, and some fruit juices (see Figure 3). The amount of quercetin received from food is primarily dependent on an individual’s dietary habits [26,27]. It is also important to note that the food content of quercetin reflects variations in soil quality, time of harvest, and storage conditions. The way of cooking food also has a noticeable effect on quercetin content: for example, onions lose about 75% of their initial quercetin content after boiling for 15 min, 65% after microwaving and 30% after frying. Recent works describe the role of this molecule against chronic diseases, such as type 2 diabetes by stimulating insulin secretion [28,29,30,31].

**Figure 4:**
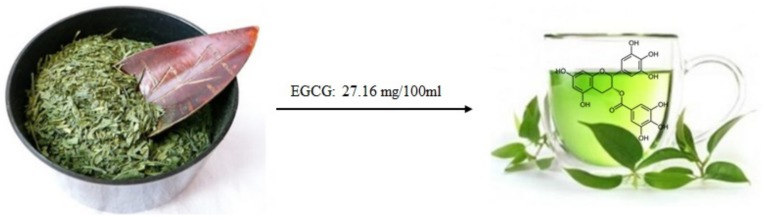
Epigallocatechin-3-gallate (EGCG) from green tea, according to the database Phenol-Explore (http://phenol-explorer.eu/).

Epigallocatechin-3-gallate (EGCG) is the most studied molecule of the flavanol class. It is a catechin conjugated with gallic acid and is abundant in green tea (see Figure 4) [32] and cocoa based products [33]. The main dietary sources of catechins, determining the intake, in Europe and USA are cocoa products, tea, and pome fruits [34]. Cocoa has the highest content of catechins, followed by prune juice and broad bean pod. Moreover, açaí oil, obtained from the fruit of the açaí palm in the form of (−)-epicatechin and (+)-catechin, is present in argan oil [35]. It was reported that green tea consumption has been correlated with a low incidence of chronic cardiovascular disease [36]. In any case, compared to other catechins found in tea, EGCG possesses the most important biological activities on inhibition of angiogenesis and, therefore, cancer progression likely due to its galloyl moiety [37,38].

#### 2.1.4. Stilbenes

**Figure 5:**
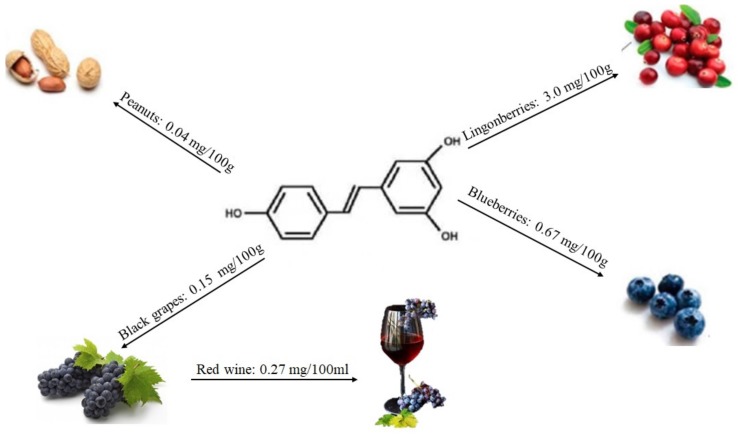
Structure of resveratrol and its content in main foods, according to the database Phenol-Explore.

Low quantities of stilbenes are present in the human diet, and the main representative is resveratrol. This compound was first isolated from the roots of *Veratrum grandiflorum* O. Loes in 1940 and later from the roots of *Polygonum cuspidatum* [39]. It is produced by plants in response to infection by pathogens [40,41,42] or to a variety of stress conditions. It has been detected in more than 70 plant species, including grapes, berries, and peanuts (see Figure 5). Several studies highlighted that this compound has a wide range of biological activities [43,44].

#### 2.1.5. Curcumin

**Figure 6:**
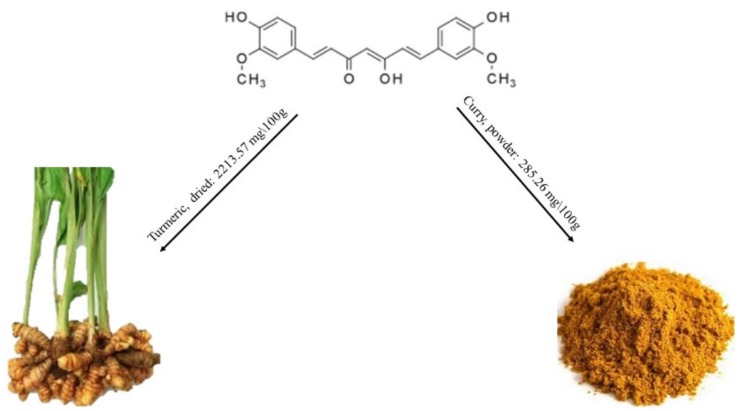
Structure of curcumin and its content in main foods, according to the database Phenol-Explore.

Curcumin is the most widely studied among curcuminoids. It is a natural phenolic that is responsible for the yellow color of turmeric (*Curcuma longa*), a member of the ginger family, Zingiberaceae. The most common applications are as an ingredient in dietary supplements, in cosmetics, and as flavoring for foods, such as turmeric-flavored beverages in South and Southeast Asia. As a food additive for orange–yellow coloring in prepared foods, its E number is E100 in the European Union. Curcumin is not very widespread in food, in fact the foods richest in curcumin are only turmeric plant and curry powder (Figure 6) [45].

### 2.2. Chemistry

Polyphenols represent the most abundant compounds among the secondary metabolites produced by plants, with more than 8000 identified compounds, ranging from small molecules such as phenolic acids to highly polymerized substances such as tannins [46]. They are produced via the phenyl propanoid pathway in which phenylalanine represents the starting compound [47]. From a chemical point of view, polyphenols are characterized by the presence of one or more aromatic rings bearing one or more hydroxyl groups. An early classification was first suggested by Manach and colleagues [48], who distinguished four classes of polyphenols, namely: 1) phenolic acids, 2) flavonoids, 3) stilbenes, and 4) lignans. Phenolic acids can be further sub-divided into two classes: hydroxybenzoic and hydroxycinnamic acids (see Table 1). These compounds exist in the free form as well as in the esterified form. Caffeic acid is most often conjugated with quinic acid to form chlorogenic acid, which is the major phenolic compound in coffee, while ferulic acid is abundantly present in cereals where it is esterified to hemicelluloses in the cell wall [49].

**Figure 7:**
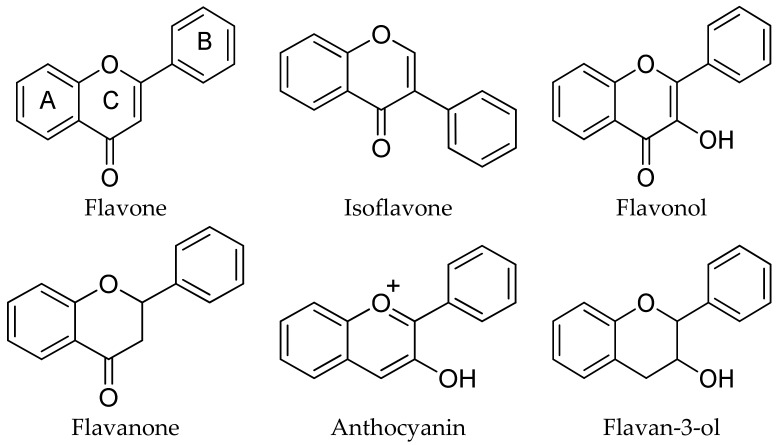
Basic structure and classification of the most important flavonoids.

The flavonoid family represents the largest class of polyphenols. The basic flavonoid structure contains two aromatic rings (labeled A and B) connected by a C3 linkage which is normally incorporated into another ring (labeled C) [50]. Flavonoids are sub-divided into six main subgroups: flavones, isoflavones, flavonols, flavanones, anthocyanins, and flavan-3-ols, according to the oxidation state of the central C ring. Structural variation in each subgroup depends on the number and position of hydroxyl and methoxyl groups. Moreover, these compounds exist in their free form, as well as in the esterified, prenylated, and glycosylated forms (see Figure 7) [51,52].

Quercetin usually occurs in plants as glycosides, linked with various sugar moieties, mostly glucose, but also galactose, rhamnose, and others. Flavan-3-ols can be found in their esterified forms, linked to a gallate moiety, as in the case of epigallocatechin-3-gallate (EGCG). Anthocyanins (from the Greek ‘anthos’, flower) are responsible for the orange, red, blue, and purple colors of flowers and fruits of many plants. They are the glycosylated form of the corresponding anthocyanidins (aglycones) [52].

Tannins represent an important group of polymeric phenolic compounds and are usually divided into two subgroups: hydrolyzable tannins and condensed tannins. Hydrolyzable tannins contain a central core of glucose esterified with gallic acid moieties (gallotannins), or with hexahydroxydiphenic acid (ellagitannins). These compounds have a molecular weight ranging from 2000 to 5000 Daltons. Condensed tannins, also referred to as proanthocyanidins, are oligomers or polymers of flavan-3-ols linked through an interflavan carbon bond [49].

**Figure 8:**
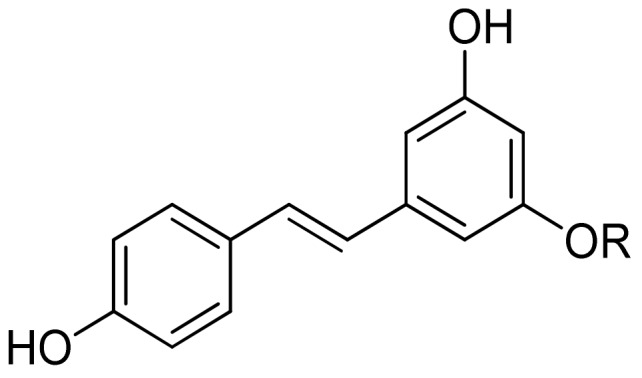
Structure of resveratrol (R = H) and resveratrol glucoside (R = glucose).

Stilbenes and lignans are less common plant phenolics. Resveratrol (3,4′,5-trihydroxystilbene, see Figure 8) is the most investigated compound belonging to the stilbene class existing in cis and trans forms. It is predominantly found in grapes and grape juice as trans-resveratrol glucoside (trans-piceid).

**Figure 9:**
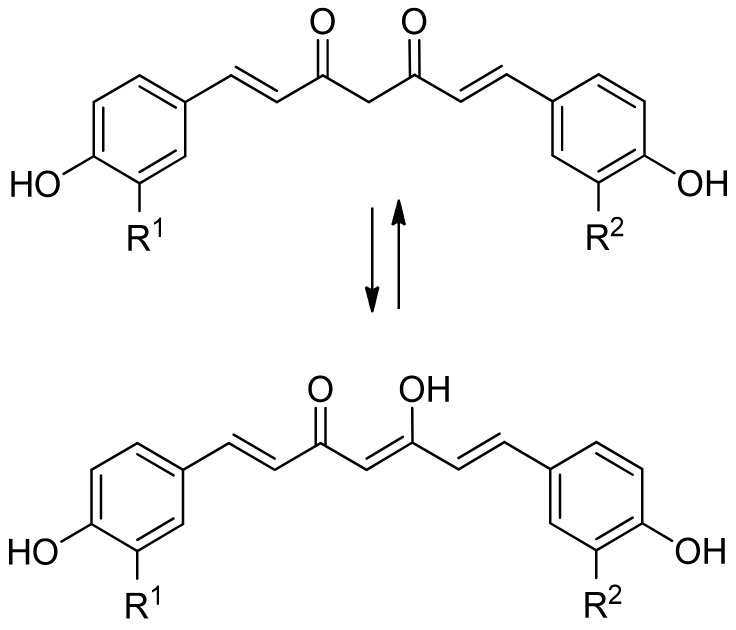
Structure and keto–enol tautomerism of curcumin (R^1^ = R^2^ = OMe), desmethoxycurcumin (R^1^ = OMe, R^2^ = H), and bisdesmethoxycurcumin (R^1^ = R^2^ = H).

Curcumin [1,7-bis(4-hydroxy-3-methoxyphenyl)-1,6heptadiene-3,5-dione] is a non-polar polyphenol. It is a bis-α,β-unsaturated β-diketone and exists in different tautomeric forms (see Figure 9). The β-diketone form prevails in acidic and neutral aqueous solutions, while the enol form predominates in more alkaline media [53]. In the case of curcumin, the aromatic rings are functionalized with hydroxy and methoxy groups, whereas the other curcuminoids lack one or both methoxy groups (desmethoxycurcumin and bisdesmethoxycurcumin, respectively).

### 2.3. Nutritional Supplements

Following the promising result from in vivo and in vitro studies, over the last decade, there was a strong development of nutritional supplements based on polyphenols. However, strong and solid studies on human efficacy are still lacking. Despite that, quercetin, EGCG, curcumin, and resveratrol are marketed as dietary supplements. These molecules have been recognized as GRAS (generally recognized as safe) by the Food and Drug Administration (FDA) as well as by the European Food Safety Authority (EFSA) for the beneficial effects on the protection of DNA, proteins, and lipids from oxidative damage, highlighting their antioxidant power. 

Quercetin is used as an ergogenic supplement (a supplement that could improve sports performance) but the results on this capability are controversial. For example, Neiman and coworkers supplemented quercetin to cyclists for two weeks (1 g per day) and analyzed muscular biopsy finding a slightly significant improvement in mitochondrial density [54]. On the other hands, meta-regression analysis relative to subjects’ fitness level and plasma quercetin concentration achieved by supplementation was not significant [55]. The point of view of Kerksick and the International Society of Sport Nutrition is that quercetin is safe and is a good antioxidant, but it needs more studies to evaluate its ergogenic power [56].

EGCG from green tea and green tea extracts is widely used in traditional Chinese medicine, so it is considered safe even in huge dosages (>1–3 g per day) it can be a pro-oxidant bringing negative effects such reactive oxygen species (ROS) production [54,57]. Despite this dichotomic effect, to date no studies confirmed the negative effect of EGCG in humans. In contrast, an interesting study performed by Pervin and coworkers showed that green tea consumption leads to an improvement in cognitive function [58]. Recently, Xicota and co-workers showed that EGCG has modest beneficial effect on weight management in Down syndrome subjects and cognitive function [59]. Furthermore, EGCG has also been seen to have a sex-dependent effect on lipid profile that was related to changes in body mass and composition.

Curcumin is widespread in many Asian cuisines as well as in Ayurvedic medicine [60]. Besides that, the antioxidant and anti-inflammatory properties of curcumin are well known [61], dealing with various pathologies such arthritis and osteoarthritis [62,63,64,65,66,67], obesity and diabetes [68,69]. In addition to its very low bioavailability, the real mechanism of action of curcumin is still uncertain. It was speculated that it probably acts via the “sanitation” of the gut and the consequent healing of inflammation. Therefore, it was supposed that the microbiome could yield active metabolites from curcumin. However, the mechanisms of action remain unclear; it probably possesses an epigenetic modulating power but to date only in vitro studies are in support of these important effects [70].

In the 1992, Renaud and co-workers published an interesting study, pointing out what is known as the “French paradox” [71]. The low susceptibility of French people to CVD linked to the use of red wine. Red wine and obviously grape are a source of resveratrol, a powerful antioxidant with benefits for muscle strength with anti-inflammatory effects [72,73]. Resveratrol is able to regulate metabolism [74], and it is useful in the treatment of neurodegenerative diseases [75], diabetes [76], cardiovascular diseases [77,78,79], and cancer [80]. In humans, a dose of 450 mg per day is considered safe [81].

### 2.4. MicroRNAs

Used as nutritional supplements, polyphenols are able to influence the activity of transcription factors or to modulate microRNAs (miRNAs). miRNAs are defined as small noncoding RNA molecules, having from 21 to 22 nucleotides. Their synthesis proceeds through well-defined biochemical steps: the primary transcripts also called hairpin-shaped (known as pre-miRNAs) are derived from: i) introns of their corresponding transcription parts and ii) intergenic regions of DNA, catalyzed by RNase II; the catalytic cleavage of the pre-miRNAs generates smaller transcripts, called pre-miRNAs about 70 nucleotides long [82]. This biochemical step is catalyzed by the polymerase III Drosha. Then, DGCR8 a protein as a dsRNA (double strand-RNA) binding molecule makes a complex with Drosha, forming a “microprocessor” able to cut the initial transcript to a 70-nucleotide length with an incomplete stem-loop structure, called pre-miRNA. The pre-miRNAs are a substrate that easily proceed to the cytoplasm through the carrier Exportin 5 [83]. Then, a specific helicase, known as Dicer RNase, together with a second dsRNA binding protein, called TAR-RNA binding protein (TRBP) performs the last cleavage process of pre-miRNAs in their hairpin site and converts them to small double-stranded RNAs, containing the mature miRNA and its complementary strand.

TRBP is able to recruit the argonaute (AGO) protein as the main factor for RNA-induced silencing complex (RISC) loading [83]. Finally, mature miRNAs are packaged into exosomes for extracellular and bloodstream transport [84].

Their effect is recognized after specific binding to complementary nucleotides of the seed region (about 2–8 nucleotides long) of target mRNA suppressing its translation and stability [85]. Recently, the role of nutrition and microRNAs as powerful regulators metabolic functions and the maintenance of oxidative stress is emerging [86,87,88]. Since dietary factors may have an influence on miRNA biogenesis, it seems reasonable to assume that some bioactive compounds present in different foods may modulate the development and progression of certain diseases. Therefore, bioactive compounds present in foods may affect endogenous miRNA synthesis.

It was demonstrated that natural compounds such as polyphenols can stimulate tumor suppressor genes, altering miRNA expressions. In this regard, several in vitro studies show the effect of quercetin, EGCG, curcumin, and resveratrol on miRNA expression in model systems. Currently, many investigations have focused on modifying the miRNA functions in cancer cells to achieve therapeutic approaches. In addition, in the case of resveratrol, a human study has also been carried out. At the moment of the writing of this article, 2675 human mature miRNA sequence have been described in miRBase 22 (http://www.mirbase.org/).

#### 2.4.1. Quercetin and MicroRNAs

The protective effects of quercetin on human health are mediated by multifaceted, pleiotropic action even from an epigenetic point of view. Currently, much research has focused on modulating the miRNA expression in cancer cells in order to achieve therapeutic approaches. Quercetin per se, was able to increase the expression of miR-146a in human breast cancer cells [89]. Similar results were achieved by quercetin derivatives on miR-146a in lipopolysaccharide (LPS)-treated normal colon cells, as well as in murine macrophages in which miR-155 expression was decreased [90]. The upregulation of miR-146a induced Toll-like receptor 4 (TLR4) stimulation which regulates nuclear factor kappa-light-chain-enhancer of activated B cells (NF-κB) and other TLR mediators of inflammation [91].

The influence of quercetin was also studied in several types of human cancer cell lines pointing out the upregulation of let-7a, let-7c, miR-200b-3p, and miR-142-3p in pancreatic ductal adenocarcinoma [92,93,94,95]. The upregulation of mir-let-7 family function as inhibitor molecules able to target *K-Ras* gene and therefore affects proliferation functioning as biomarkers for both prognosis and therapy for precision medicine in cancer [92,96]; while miR-200b-3p and miR-142-3p regulates the mode of self-renewing divisions and the heat shock protein 70, respectively [92,93,94,95].

Furthermore, miR-16, miR-217, and miR-145 were modulated by quercetin in lung adenocarcinoma, osteosarcoma, and ovarian cancer cells, respectively [97,98,99]. In the lung, miR-16 was able to downregulate Claudin-2 which is also a mediator of leaky gut barrier during intestinal inflammation [97,100]. On the other hand, miR-217 enhances cisplatin sensitivity interfering with *K-Ras* pathways [98] and miR-145 inhibits and control targeting genes that have similar behavior in apoptosis and in different Gene Expression Omnibus (GEO) databases [99,101].

#### 2.4.2. EGCG and MicroRNAs

Similar to other polyphenols, EGCG was extensively studied in inflammation. In particular, when EGCG is used in interleukin-1-beta (IL-1β)-stimulated human osteoarthritis chondrocytes cell line, it was able to upregulate and downregulate a plethora of miRNAs. Especially, let-7 family (let-7a-5p, let-7b-5p, let-7c, let-7d-5p, let-7f-5p, let-7i-5p), miR-140-3p, miR-193a-3p, miR-199a-3p, miR-27b-3p, miR-29a-3p, miR-320b, miR-34a-5p, miR-3960, miR-4284, miR-4454, miR-497-5p, miR-5100, and miR-100-5p were upregulated [102]. It is of note that miRs exosome cargo is today proposed in the osteoarthritis management [103]. With regard to this, recent studies showed that the exosomes derived from mesenchymal stem cells maintain chondrocyte homeostasis, ameliorating the pathological severity of the disease this is due to the miR-100-5p which in turns inhibits the mTOR-autophagy pathway [104]. In the same set of experiments, researcher documented that EGCG was able to decrease let-7e-5p, miR-103a-3p, miR-151a-5p, miR-195-5p, miR-222-3p, miR-23a-3p, miR-23b-3p, miR-26a-5p, miR-27a-3p, miR-29b-3p, miR-3195, miR-3651, miR-4281, miR-4459, miR-4516, miR-762, and miR-125b-5p expression [102]. The decrease of this latter miR is not positive for the prevention of cartilage breakdown in osteoarthritis [105].

Since modulation of miRNA expression in cancer cells could be a therapeutic strategy, EGCG’s biochemical effects were also studied in cancer. EGCG was able to upregulate miR-140-3p and miR-221 in melanoma and hepatoma cell lines, respectively [106,107] inhibiting osteopontin in induced liver fibrosis [107]. Similar results for miR-140-3p were obtained under EGCG treatment in chondrocyte [102]. Furthermore, EGCG induced the increase of miR-3663-3p, miR-1181, miR-3613-3p, miR1281, and miR-1539 and the decrease of miR-221-5p, miR-374b, miR-4306, miR-500a-5p, and miR590-5p in human dermal papilla cells [108] from scalp hair. The sensitivity of the scalp is also higher in migraine, and this latter miR was found downregulated in humans suffering from pain-migraine. The level of this miR-590-5p was restored with diet [109]. Interestingly, green tea is recommended to relieve migraine attack frequency [110].

#### 2.4.3. Curcumin and MicroRNAs

Curcumin treatment on schwannoma cells by miRs array revealed that miR-350, miR-17-2-3p, let 7e-3p, miR-1224, miR-466b-1-3p, miR-18a-5p, and miR-322-5p were downregulated while miR-122-5p, miR-3473, miR-182, and miR-344a-3p were upregulated. This latter miRs have a role in the control of apoptosis in schwannoma cells [111]. In addition, in several types of cancer, curcumin has been shown to play a role in the modulation of miRs controlling apoptosis, i.e., miR-33b and miR-205-5p are upregulated in melanoma cancer cells while miR-21 was downregulated. On the contrary, in colorectal cancer, upon treatment with curcumin, miR-21, miR-3a/c, and miR-27a were upregulated, and miR-200b/c, miR-141, miR-101, miR-429, and miR-34a displayed lower expression with respect to the untreated cells [112].

Upregulation of the cluster miR-192-5p/215 was reported in lung cancer upon curcumin treatment as well as miR-9, miR-205, miR-200a/b, miR-15a/16-1, and miR-203 in ovarian, prostate, hepatocellular, leukemia, and bladder cancer cells, respectively. In breast cancer cells, miR-19 and miR-15a/16-1 were found upregulated, whereas miR-34a and miR-181b were downregulated. In thyroid carcinomas miR-21 and miRNA-200c were found upregulated while let7c, miR-26a, miR-215, miR-192-5p, and miR-125b were downregulated [112]. The latter was also found downregulated upon curcumin treatment in nasopharyngeal carcinoma [112].

Furthermore, curcumin induced apoptosis in cisplatin-resistant human ovarian cancer cells through caspase-3 activation and poly (ADP-ribose) polymerase (PARP) cleavage, via upregulation of miR-9 [113].

Altogether the miRs modulated by the polyphenols discussed here resulted to influence several pathways including Wnt and mitogen-activated protein kinase (MAPK) signaling, pathways in cancer and basal cell carcinoma, as well as in adherence junction, neurotrophin signaling, and axon guidance, and, last but not least, cytokine–cytokine receptor interaction as it is present in KEGG (Kyoto Encyclopedia of Genes and Genomes) database (https://www.genome.jp/kegg/pathway.html).

#### 2.4.4. Resveratrol and MicroRNAs

Currently, more than a hundred scientific documents have confirmed that the effect of resveratrol in the prevention or treatment of various diseases, including cancer, is mediated by miRs. The biological effects of resveratrol were studied in human colon cancer in which it significantly decreased the levels of miR-17, miR-21, miR-25, miR-92a-2, miR-103-1, and miR-103-2. Those miRs in certain contexts have been shown to act as oncomiRs [114]. In prostate cancer [115] and in melanoma, resveratrol decreased miR-221 levels [116]. While in lung tumors, resveratrol led to an upregulation of miR-200c [117]. In addition, it acts to decrease miR-542-3p and increase miR-122-5p in estrogen-responsive and triple-negative breast cancer cells, while only mir-122-5p is increased in the triple-negative cells [118]. Resveratrol showed effectiveness via miRs not only in aberrant pathophysiology such as cancer, but in physiological cell systems such as white adipose cell lines also resveratrol induced the expression of miR-539-5p inhibiting de novo lipogenesis [119].

In primary human fibroblasts, resveratrol was able decrease miR-566 and miR-23a, restoring mitochondrial fatty acid β-oxidation rates in primary human fibroblasts form patients harboring carnitine palmitoyltransferase-2 mutation associated with two different phenotypes (neonatal lethality or myopathy in mild forms) [120]. Therefore, resveratrol, independently of the disease, led to miR-566 and miR-23a modulation specifically [120]. Microarray analysis showed that human THP-1 monocytic cells treated with resveratrol increased the expression of miR-663 decreasing miR-155 [121]. On the other hand, the reduction of proliferation and differentiation of pre-adipocytes due to resveratrol treatment, led to the over expression of miR-155 [122].

Although of interest, the in vitro studies conducted so far have not been translated to humans yet. Only resveratrol has been studied in human subjects [81]. The daily intake used was one capsule/day of grape extract (139 mg) containing resveratrol (8.1 mg) by men with T2D, hypertension, and BMI > 30 kg/m^2^ for six months and two capsules/day for further six months. This treatment yielded the upregulation of miR-21, miR-181b, miR-663, and miR-30c and the concomitant lower levels of inflammatory cytokines such as IL-6, chemokine (C-C motif) ligand 3 (CCL3), IL-1β, and tumor necrosis factor α (TNF-α). Additionally, miR-155 increased as well in peripheral blood mononuclear cells. The increase in these miRNAs was associated with a reduction of inflammation mediated by the regulation of the TLR and NF-kB pathways and inflammatory cytokine gene expression [81].

### 2.5. Pharmacokinetic Profile

Various reports unveiled polyphenols as promising therapeutic agents owing to their broad spectrum of biological activities. These compounds have long been recognized to possess free radical scavenging properties, however, the presence of both hydrophobic and hydrophilic domains within the chemical structure enables polyphenols to affect membrane dynamics through the arrangement of membrane proteins and the formation of functional complexes responsible for cell signal transduction and the regulation of the metabolism [123]. This mechanism of action underlies most of the beneficial effects of polyphenols, however the effectiveness of these compounds in disease prevention and human health improvement is tightly related and limited to their bioavailability [48]. The concept of bioavailability encompasses several variables such as intestinal absorption, metabolism by gut microbiota, intestinal and liver metabolism, biological properties of metabolites, distribution at tissues level, and excretion, which in turn depend upon the chemical structure of xenobiotics.

The various chemical forms of polyphenols lead to high variability in their rate and extent of intestinal absorption as well as in the nature of circulating metabolites [48]. Most of these compounds are in the glycosylated form resulting in a low grade of absorption of their native molecule. Commonly flavonoids show as sugar moiety glucose or rhamnose, and following their ingestion, these compounds undergo deglycosylation prior to being absorbed [124]. Hydrolysis of saccharide moiety occurs at the level of gastrointestinal cells, and it is carried out by intracellular cytoplasmic β- glucosidase (CBG) [125], of note, the expression pattern is tissue specific and often regulated during development. In humans, different glycosidases have been documented: i) lactase phlorizin hydrolase (LPH) and CBG at the level of red blood cells and cytosol, respectively. Both enzymes hydrolyzed glycosylated flavonoids in the more hydrophobic aglycones, thus promoting passive diffusion through enterocytes [126,127,128]. However, determined glycosylated flavonoids, such as quercetin-4′-glucoside, were found to be also actively transported into enterocytes through the active sodium-dependent glucose transport (SGLT1) [128]. It is worth mentioning that flavonoids with rhamnose moiety are not substrates for human β-glycosylases being cleaved by colon microflora α-rhamnosidases before the absorption process [129]. A large proportion of polyphenols is constituted by flavan-3-ols such as (−)-epicatechins. These compounds are never glycosylated but often acetylated by gallic acid. As revealed by pharmacokinetic studies, catechins, and particularly EGCG, are predominantly absorbed in the jejunum and the ileum, via a paracellular diffusion through epithelial cells without any de-conjugation or hydrolysis [130].

An important step limiting the absorption of the determined flavonoids is represented by intestinal efflux [131]. This process is affected by membrane transporters and, among these, members of the adenosine triphosphate (ATP)-binding cassette (ABC) superfamily such as multidrug-resistance protein (MRP), P-glycoprotein (P-gp), and breast cancer resistance protein (BCRP) have been reported to be involved in the regulation of some flavonoids intestinal efflux and ultimately to influence the net amount that is absorbed into systemic circulation [132,133]. The efflux of quercetin and epicatechin metabolites is thought to occur by MRP2, located on the luminal side of epithelial cells [131] while the monocarboxylate transporter P-gp, MRP1, and MPR2 play significant roles in the cellular accumulation and possible effects of (−)-epicatechin gallate [134]. Since ABC transporters are ubiquitously present in most tissues, the interplay between flavonoids and ABC transporters could not only modulate the extent of intestinal efflux and bioavailability but also the distribution of flavonoid conjugates to the target sites and their elimination. Moreover, bioavailability of these compounds may be amplified or reduced by a selective interaction with ABC transporters [135] when co-administered.

Both quercetin and EGCG undergo to extensive metabolism at both enterocytes and liver levels by glucuronidation, sulfation, and methylation reactions [136,137,138]. Some of the liver conjugates are excreted as bile components and undergo enterohepatic recirculation. The de-conjugated compounds are then regenerated by gut microbial enzymes before being reabsorbed again [139,140,141,142] while the unabsorbed metabolites are eliminated via feces. All the conjugation mechanisms are highly efficient, therefore considerable amounts of metabolites reach the bloodstream [143]. These products retain the biological activity producing similar, stronger, or weaker effects compared with parent compounds [144,145]. An exception is green tea catechins, whose aglycones constitute a substantial proportion of the total amount in plasma, as they are devoid of the sugar moiety and, hence, quickly absorbed at the small intestine without further modifications [146].

The poor systemic bioavailability also affects the mechanism of action across conditions and doses of resveratrol as demonstrated by the in vivo non-reproducibility of in vitro effects [147,148]. In humans, resveratrol is highly absorbed orally. However, a rapid and extensive biotransformation of the polyphenol occurs after the absorption phase into the enterocytes. Specifically, resveratrol undergoes sulfation and glucuronidation mediated by sulfotransferase 1A1 (SULT1A1) and UDP glucuronosyltransferase 1 family, polypeptide A1 (UGT1A1) and UDP glucuronosyltransferase 1 family, polypeptide A9 (UGT1A9) enzymes, respectively. The metabolism takes place in multiple organs and cell types, and the observed biotransformation differs in metabolite levels [149], according to tissue expression of the specific enzymes involved in the biotransformation [150]. Of note, there is an inter-species variation of phase II metabolism and, in this respect, resveratrol sulfates are the main conjugates in humans, while glucuronides conjugates are dominant in pigs and rats [151]. It is worth mentioning that, drug metabolism is a well-documented cause of inter-individual variability and for both SULTs and UGTs genetic polymorphisms have been reported [152,153]. After absorption and conjugation, resveratrol sulfates and glucuronides through the ABC transporters expressed at both apical and basolateral portions of enterocyte membrane can be transported either in the intestinal lumen or in the bloodstream where they bind to lipoproteins or albumin before distributing to peripheral tissues [154]. Of note, transporters are not limited to participating in the absorption and distribution of resveratrol and metabolites in the duodenum and jejunum, as they are also expressed in other tissues, such as kidneys [155,156,157,158], thus contributing to excretion processes. Unlike resveratrol, the low availability of curcumin in humans after oral intake is primarily due to a low grade of absorption by the small intestine coupled with fast metabolism and elimination.

The poor bioavailability is also intensified by the curcumin’s capability to bind to enterocyte proteins that can modify its structure [159,160,161]. The liver is the primary site of phase I and II curcumin biotransformation along with intestine and gut microbiota [162]. Extensive metabolic reduction also occurs at enterocyte and hepatocyte levels leading to the formation of dihydrocurcumin, tetrahydrocurcumin, hexa-hydrocurcumin, and octahydrocurcumin which in turn are converted through conjugation into physiologically inactive constituents [163]. Of note, these reduced compounds can exist both in free form or as glucuronides [162]. Phase II metabolism takes place in the intestinal and hepatic cytosol on both curcumin and its phase I products by conjugation with glucuronic acid or sulfation at phenolic site. Curcumin is sulfated by SULT1A1, SULT1A3 in the cytosolic fraction while UGTs catalyze the glucuronidation at the level of hepatic microsomes. Gut microflora also contribute to curcumin metabolism mainly through sulfation or demethylation reactions leading to tetrahydrocurcumin [164] and demethylcurcumin and bis-demethylcurcumin [165], respectively. Of note, curcumin metabolites retain all pharmacological properties of the parent compound showing anti-oxidant, anti-inflammatory, antitumor, cardioprotective, and anti-diabetic effects [5,19,20]. Gender can also significantly affect curcumin pharmacokinetics. The differences are related to gender-specific factors, among these, a higher activity of hepatic drug efflux transporters in men and the presence of higher body fat in women [166,167].

## 3. Methods

PubMed, Embase, Cochrane library, and reference lists were searched for articles published until 1 November 2019, using the keywords “polyphenols”, “radical oxygen species and antioxidant activity”, “inflammatory biomarkers”, “epidemiology”, “food source”, “gene expression”, “microRNAs”, “microRNAs and inflammatory biomarkers”. Secondary searches included articles cited in sources identified by the previous search.

## 4. Conclusions

Understanding polyphenol consumption is essential to determine the nature and the distribution of these compounds in our diet. Despite their poor bioavailability, quercetin, EGCG, curcumin, and resveratrol are marketed as supplements. Only for the latter one, studies were conducted in human subjects pointing out its capability to influence miRNA expression pattern related to inflammation. In this view, as a future direction, we suggest using the microRNAs linked to inflammatory, antioxidant, or immune status as marker(s) for monitoring nutraceutical effects of polyphenols. At the moment, polyphenols are becoming protagonists in the nutraceutical scenario without studies on human subjects. The main factor responsible for this delay is the variety and the complexity of their chemical structure and to some extent their gut microflora metabolite.

## Figures and Tables

**Table 1:** Structure and classification of phenolic acids.

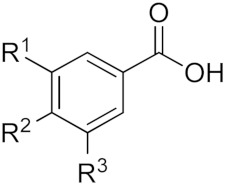 Hydroxybenzoic acids	**Name**	**R^1^**	**R^2^**	**R^3^**
	*p*-Hydroxybenzoic acid	H	OH	H
	Protocatechuic acid	OH	OH	H
	Vanillic acid	OCH_3_	OH	H
	Gallic acid	OH	OH	OH
	Syringic acid	OCH_3_	OH	OCH_3_
			
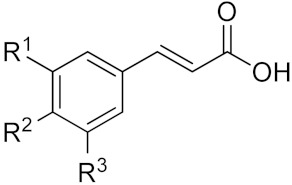 Hydroxycinnamic acids	**Acid**	**R^1^**	**R^2^**	**R^3^**
	*p*-Coumaric acid	H	OH	H
	Caffeic acid	OH	OH	H
	Ferulic acid	OCH_3_	OH	H
	Sinapic acid	OCH_3_	OH	OCH_3_
